# High-yield production of 1,3-propanediol from glycerol by metabolically engineered *Klebsiella pneumoniae*

**DOI:** 10.1186/s13068-018-1100-5

**Published:** 2018-04-09

**Authors:** Jung Hun Lee, Moo-Young Jung, Min-Kyu Oh

**Affiliations:** 1 Department of Chemical and Biological Engineering, Korea University, Seongbuk-gu, Seoul, 02841 Republic of Korea; 2 CJ Research Institute of Biotechnology, Suwon, Gyeonggi 16495 Republic of Korea

**Keywords:** 1, 3-PDO, *Klebsiella pneumoniae*, Glycerol, Byproducts, Glycerol assimilation pathway, Co-substrate, Mannitol, *dha* operon

## Abstract

**Background:**

Glycerol is a major byproduct of the biodiesel industry and can be converted to 1,3-propanediol (1,3-PDO) by microorganisms through a two-step enzymatic reaction. The production of 1,3-PDO from glycerol using microorganisms is accompanied by formation of unwanted byproducts, including lactate and 2,3-butanediol, resulting in a low-conversion yield.

**Results:**

*Klebsiella pneumoniae* was metabolically engineered to produce high-molar yield of 1,3-PDO from glycerol. First, the pathway genes for byproduct formation were deleted in *K*. *pneumoniae*. Then, glycerol assimilation pathways were eliminated and mannitol was co-fed to the medium. Finally, transcriptional regulation of the *dha* operon were genetically modified for enhancing 1,3-propanediol production. The batch fermentation of the engineered strain with co-feeding of a small amount of mannitol yielded 0.76 mol 1,3-PDO from 1 mol glycerol.

**Conclusions:**

*Klebsiella pneumoniae* is useful microorganism for producing 1,3-PDO from glycerol. Implemented engineering in this study successfully improved 1,3-PDO production yield, which is significantly higher than those reported in previous studies.

**Electronic supplementary material:**

The online version of this article (10.1186/s13068-018-1100-5) contains supplementary material, which is available to authorized users.

## Background

Biodiesel production has increased significantly over the past decade and, along with it, the production of glycerol, a byproduct of biodiesel production, has also increased. Intensive studies have been performed for adding value on glycerol [1]. Glycerol can be converted into a number of valuable products, including 1,3-propanediol (1,3-PDO), 3-hydroxypropionic acid, 2,3-butanediol (2,3-BDO), and lactate by metabolic engineering [2]. Among these, 1,3-PDO is a valuable chemical product that can be used as a solvent in the cosmetic industry and as a monomer to synthesize biopolymers. In 2012, the international demand for 1,3-PDO was 60.2 kton, and this has been expected to grow by 150% until 2019 [3]. Among the chemical and biochemical processes for 1,3-PDO production, fermentation has several advantages over chemical processes, including a requirement for moderate reaction conditions [4, 5].

Certain microorganisms, including *Klebsiella*, *Citrobacter*, *Enterobacter*, *Bacillus*, *Lactobacillus,* and *Clostridium,* are natural 1,3-PDO producers and have genes that encode enzymes for the required reactions on their chromosomes. Among the natural producers, *Klebsiella pneumoniae* in particular has advantages. This species grows fast on glycerol under both aerobic and anaerobic conditions, and a number of genetic modification tools have been already established. In addition, *K. pneumoniae* produces vitamin B_12_, a coenzyme of glycerol dehydratase, which is involved in synthesis of the 1,3-PDO precursor, 3-hydroxypropionaldehyde [6]. For these reasons, *K. pneumoniae* has often been used as a host for the biochemical production of 1,3-PDO from glycerol. 1,3-PDO production has been increased by various strategies such as preventing byproduct formation, co-producing valuable metabolites, and rebalancing reducing cofactors [7–10]. The titers, productivities, and yields of 1,3-PDO from glycerol achieved in recent studies are summarized in Table 1. When producing 1,3-PDO using microorganisms, many unwanted byproducts, such as lactate and 2,3-BDO, are produced. In particular, the separation of 1,3-PDO from 2,3-BDO significantly increases the overall cost of the process [11]. Therefore, generating a higher molar yield of 1,3-PDO from glycerol is highly desirable [7].

**Table 1:** Comparison of the recent studies for 1,3-propanediol production from glycerol using engineered *Klebsiella pneumonia*

Sources	Fermentation Time (h)	Titer (g L^−1^)	Yield (mol mol^−1^ glycerol)	Productivity (g L^−1^ h^−1^)	Comments
Lin et al. [10]	30	76.80	0.66	2.56	Fed-batch
Kumar et al. [9]	36	38.95	0.49	1.08	Fed-batch with no BDO production
Wang et al. [7]	33	86.00	0.59	2.69	Fed-batch
Xin et al. [8]	30	76.20	0.43	2.54	Fed-batch
This study	24	20.59	0.76	0.86	Batch with no BDO production

**Fig. 1:**
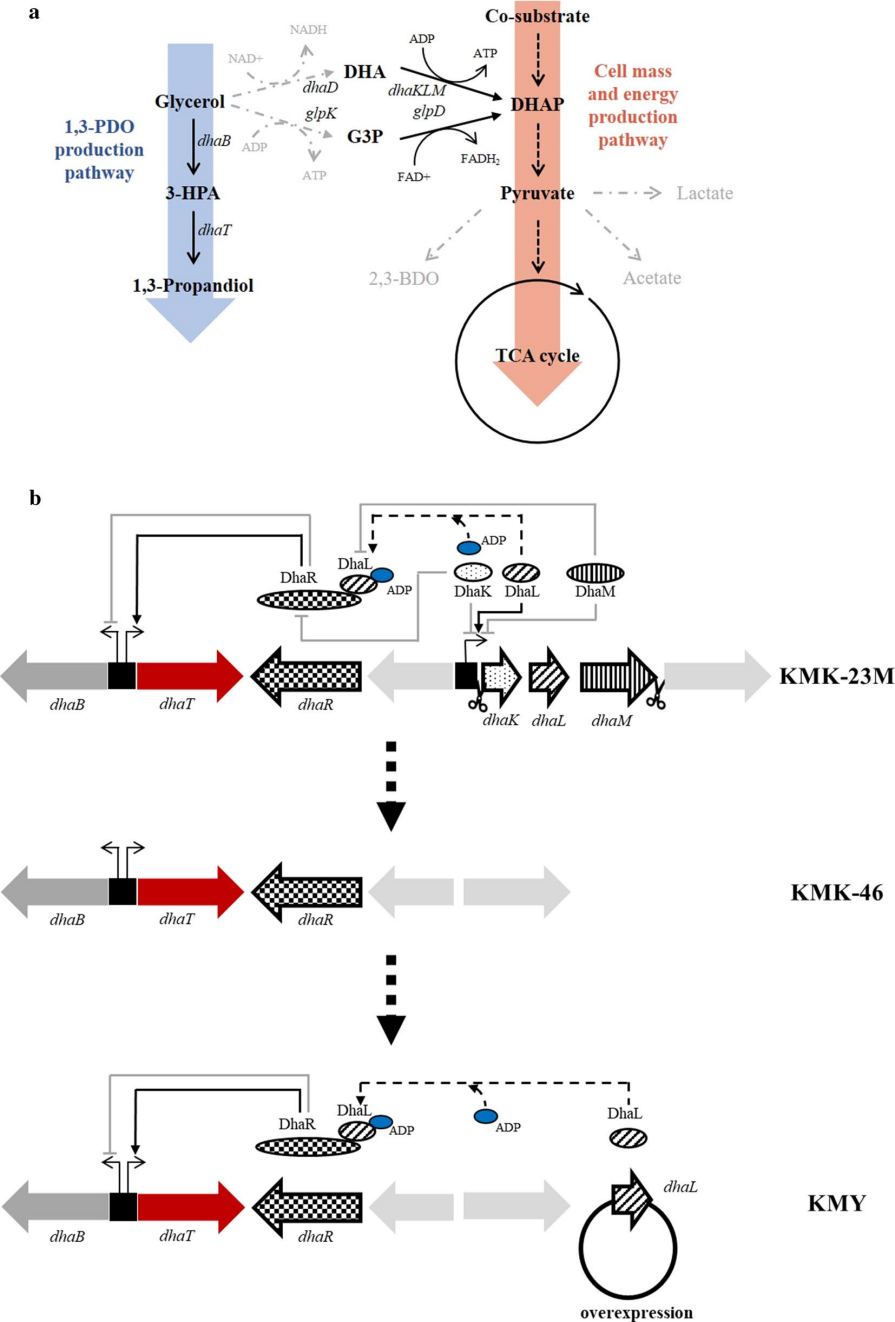
Engineered metabolic pathways and regulators in this study. **a** Metabolic pathways of *K. pneumoniae* for 1,3-propanediol (1,3-PDO) production and microbial growth. Eliminated pathways in this study on byproducts formation and glycerol assimilation are presented with dashed gray arrows. Pathways with multiple steps are presented with dotted arrows. The acronyms, 3-HPA, DHA, G3P, DHAP, and TCA, refer to 3-hydroxypropionaldehyde, dihydroxyacetone, glyceraldehyde 3-phosphate, dihydroxyacetone phosphate, and tricarboxylic acid, respectively. Genes involved in those pathways are in italics. **b** Engineering scheme of *dha* operon with its regulations. PdhaK, the promoter of *dhaK*, is transcriptionally repressed by DhaK and DhaM and activated by DhaL. ADP forms a complex with DhaL, which binds to the sensing domain of DhaR, thus activating DhaR. ADP and DhaL complexes are inactivated by DhaM-mediated phosphorylation. DhaK inactivates DhaR by binding. Activated DhaR by ADP-DhaL complex activates *dhaT* expression, while represses *dhaB*. Black solid arrows stand for activation and gray closed arrows stand for inhibition. Black dashed arrows indicate protein modification [14]. KMK-23M, KMK-46, and KMY on the right-hand side represent the engineered strains (Table 2)

In this present study, we utilized several strategies to generate a higher molar yield of 1,3-PDO from glycerol (Fig. 1). First, genes in the pathway responsible for production of unwanted byproducts were eliminated, including genes encoding lactate dehydrogenase (*ldhA*, Gene ID: 12545375 [Genbank]), formate acetyltransferase (*pflB*, Gene ID: 12544797 [Genbank]), and alpha-acetonelactate decarboxylase (*budA*, Gene ID: 12546009 [Genbank]) [12, 13]. Second, glycerol assimilation pathways were eliminated by deleting the genes encoding glycerol kinase (*glpK*, Gene ID: 12548005 [Genbank]) and glycerol dehydrogenase (*dhaD*, Gene ID: 12547488 [Genbank]) (Fig. 1a). The resulting strain had an improved 1,3-PDO molar yield from glycerol when a co-substrate, mannitol, was supplied for cell growth and maintenance. Finally, *dha* regulators were engineered for more efficient conversion of glycerol to 1,3-PDO (Fig. 1b). Negative regulators of DhaK (NCBI-Protein ID: AEJ99992) and DhaM (NCBI-Protein ID: AEJ99994) were deleted, while a positive regulator, DhaL (NCBI-Protein ID: AEJ99993), was overexpressed [14]. The resulting strain produced 1,3-PDO more rapidly during batch fermentation with a molar yield of 0.76 mol mol^−1^ glycerol. This yield was superior to those reported in other studies, which have reported molar yields of up to 0.66 mol mol^−1^ glycerol (Table 1) [10, 15, 16].

## Methods

### Strains, plasmids, and primers

The strains, plasmids, and primers used in this study are listed in Table 2 and Additional file 1: Table S1. All *K. pneumoniae* strains were derived from the wild-type strain KCTC 2242 (Korean Collection for Type Culture) [17]. KMK-01, KMK-02, and KMK-05 strains have been reported in our previous study [12]. To construct additional deletion mutants, λ red recombination was used with pRedET-transformed strains [18]. The plasmid pKD4 was used to synthesize an antibiotic-resistant gene flanked by FLP recognition target sites, while 707-FLP was used to eliminate the resistance cassette. The KMK-11, KMK-12, KMK-21, KMK-22, KMK-23, and KMK-46 strains (Table 2) were constructed using this method. Deletions of target genes were confirmed by agarose gel electrophoresis (Additional file 2: Figure S1) and sequencing their PCR products generated with genomic DNA of the strains and confirmation primers, indicated by ‘con’ in their name in Additional file 1: Table S1.

**Table 2:** *K. pneumoniae* strains and plasmids used in this study

Strain or plasmid	Description	Source
Strains		
KMK-01	KCTC 2242 Δ*wabG*	[12]
KMK-02	KCTC 2242 Δ*wabG* Δ*ldhA*	[12]
KMK-05	KCTC 2242 Δ*wabG* Δ*ldhA* Δ*pflB*	[12]
KMK-12	KCTC 2242 Δ*wabG* Δ*ldhA* Δ*pflB* Δ*budA*	This study
KMK-21	KCTC 2242 Δ*wabG* Δ*ldhA* Δ*pflB* Δ*budA* Δ*dhaD*	This study
KMK-22	KCTC 2242 Δ*wabG* Δ*ldhA* Δ*pflB* Δ*budA* Δ*glpK*	This study
KMK-23	KCTC 2242 Δ*wabG* Δ*ldhA* Δ*pflB* Δ*budA* Δ*dhaD* Δ*glpK*	This study
KMK-23M	KMK-23 edited 5′-UTR of *mtlA* for reduced expression level	This study
KMK-46	KMK-23M Δ*dhaKLM*	This study
KMY	KMK-46 harboring pZS21*dhaL*	This study
Plasmids		
pRedET	λ phage red γ,β,α-producing vector, pBAD_promoter; ori101 Tet^r^	Gene bridges
707-FLP	Flp recombinase-producing vector; Psc101 ori cI1578 Tet^r^	Gene bridges
pKD4	FRT-flanked resistance cassette-involved vector; oriRγ Km^r^	[16]
pZA31::MCS	Expression vector, Cm^R^, p15A ori	Expressys
pZS21::MCS	Expression vector, Km^R^, pSC101 ori	Expressys
pZA-Cas9	pZA31MCS, but P_LtetO-1_::*cas9*-PsacB::*sacB*	[23]
pZS-CRISPR	pZS21MCS, but P_LtetO-1_::TracerRNA-PsacB::*sacB*	[23]
pZS-CRISPR *mtlA*	pZS21MCS, but P_LtetO-1_::TracerRNA-5′UTR(*mtlA*)-PsacB::*sacB*	This study
pZS21*dhaL*	pZS21::MCS harboring *dhaL* from wild-type *K. pneumoniae*	This study
pZA31*budABC*	pZA31::MCS harboring *budABC* from wild-type *K. pneumoniae*	This study

*Ap*^*R*^ ampicillin, *Tet*^*R*^ tetracycline, *Cm*^*R*^ chloramphenicol, *Km*^*R*^ kanamycin resistance

Decreased *mtlA* (Gene ID: 12547935 [Genbank]) gene expression in KMK-23 was achieved by mutation of its 5′-untranslated region (UTR). The sequence of 5′-UTR of *mtlA* has been designed by RBS calculator [19, 20] to have 62% *mtlA* expression compared to the parental strain. The sequence 5′-TAGACAGAGTCTAACAGACCATCGAGGAACGTATG-3′, which consists of bases − 32 to − 1 from the *mtlA* translation start codon, was chosen and introduced to the 5′-UTR region using genome editing with CRISPR/Cas9 [21, 22]. The plasmids, pZA-Cas9 and pZS-CRISPR, for genome editing were used as reported in a previous study [23]. The DNA fragments, mtlA CRISPR F and R (Additional file 1: Table S1), were synthesized, annealed, and inserted into pZS-CRISPR at BsaI to generate crRNA. The resulting plasmid was designated pZS-CRISPR *mtlA*. The DNA fragments, and mtlA rescue F and R (Additional file 1: Table S1) were synthesized, annealed, and used as rescue DNA. The plasmid pZS-CRISPR *mtlA* and rescue DNA were transformed into a pZA-Cas9 containing KMK-23 strain. The genome-edited strain, KMK-23M, was confirmed by DNA sequencing.

The *dhaL* gene was cloned into the pZS21MCS plasmid using the Gibson assembly method [24, 25]. The plasmid pZS21::MCS was cut using restriction enzymes, *Hin*dIII and *Mlu*I, and assembled with amplified *dhaL* gene fragment generated with primers dhaL F and R (Additional file 1: Table S1) by NEBuilder Assembly Tool (http://nebuilder.neb.com). The resulting plasmid, pZS21*dhaL*, was transformed into KMK-46 to construct KMY. The engineered strains and plasmids were confirmed by DNA sequencing.

### Media and culture conditions

In this study, the media components used in addition to the carbon sources were as follows: 3 g L^−1^ KH_2_PO_4_, 6.8 g L^−1^ Na_2_HPO_4_, 0.75 g L^−1^ KCl, 5.35 g L^−1^ (NH_4_)_2_SO_4_, 0.28 g L^−1^ Na_2_SO_4_, 0.26 g L^−1^ MgSO_4_·7H_2_O, 0.42 g L^−1^ citric acid, 10 g L^−1^ yeast extract, 10 g L^−1^ casamino acids, 1 mL of a microelement solution (0.07 g L^−1^ ZnCl_2_, 0.1 g L^−1^ Na_2_MO_4_·2H_2_O, 0.1 g L^−1^ MnCl_2_·4H_2_O, 0.2 g L^−1^ CoCl_2_·6H_2_O, 0.025 g L^−1^ NiCl_2_·7H_2_O, 0.02 g L^−1^ CuCl_2_·2H_2_O, and 0.06 g L^−1^ H_3_BO_3_), and 1 mL of an iron solution (10 g L^−1^ FeSO_4_·7H_2_O). Strains were selected on LB medium (10 g L^−1^ tryptone, 5 g L^−1^ yeast extract, and 10 g L^−1^ NaCl) supplemented with the appropriate antibiotics, such as 50 μg mL^−1^ chloramphenicol, 100 μg mL^−1^ kanamycin, and 20 μg mL^−1^ tetracycline.

To evaluate byproducts production of KMK-01, KMK-02, KMK-05, KMK-11, and KMK-12, 55 g L^−1^ glycerol was fed as a carbon source. For the strains, which the glycerol assimilation pathway was eliminated, 40 g L^−1^ glycerol was fed as a carbon source along with 20 g L^−1^ of mannitol, glucose, xylose, or galactose. For the KMY strain, the medium was supplemented with kanamycin (100 μg mL^−1^). Cultures were incubated in 30 mL volumes from 1% (v/v) inoculums in 100-mL Erlenmeyer flasks sealed with silicon stoppers under microaerobic conditions at 37 °C with shaking at 250 rpm. Batch fermentation was performed in a volume of 1 L from 5% (v/v) inoculums in a 2.5 L bioreactor (Bio Control & System, Daejeon, South Korea). The conditions for batch fermentation were 37 °C, shaking at 150 rpm, 0.4 vvm, and pH maintained between 6.5 and 7.0, where the pH was adjusted with 5 M NaOH and antifoam 204 (Sigma, St. Louis, MO, USA) was added as needed. Dissolved oxygen was monitored by D500 OxyProbe^®^ II DO Sensor (Broadley-James Corporation, Irvine, CA, USA) during the fermentation. The cultivations were performed at least three times.

### Analytical methods

The cell density was monitored by UV visibility spectrophotometer (Shimazu UV mini 1240; Shimadzu, Tokyo, Japan) at 600 nm (OD_600_). Culture broth (1 mL) was transferred to microcentrifuge tubes and centrifuged at 13,000 rpm for 10 min at 4 °C. Supernatants were transferred to new microcentrifuge tubes and used for analysis of metabolites. To measure concentrations of 1,3-PDO, 2,3-BDO, acetate, lactate, succinate, glycerol, mannitol, glucose, xylose, and galactose, high-performance liquid chromatography (Younglin Instrument ACME-9000, Seoul, South Korea) with a Sugar SH1011 column (Shodex, Tokyo, Japan) maintained at 70 °C, an RI detector maintained at 45 °C, and 5 mM H_2_SO_4_ as the mobile phase with a flow rate of 0.6 mL min^−1^ was used. NADH and NAD^+^ assay was conducted by NAD/NADH-GloTM Assay G9071 (Promega, Madison, WI, USA) according to the manufacturer’s protocol. Each strain for the assay was cultured to the mid-log phase (OD_600_ = 1) with 30 mL working volume in 100-mL Erlenmeyer flask and harvested for the assay.

### Real-time RT-PCR

The strains were cultured in flask and harvested in early exponential phase (OD ~ 1). RNA was prepared by RNeasy Mini Kit (Qiagen, Hilden, Germany) according to the manufacturer’s protocol. Complementary DNA was synthesized by PrimeScript™ Reverse Transcriptase 2680A (Takara, Shiga, Japan). For PCR, QPCR Green Master Mix (LRox, biotechrabbit, Hennigsdorf, Germany) was used according to manufacturer’s protocol. Relative gene expression levels were measured by quantitative PCR by StepOne™ Real-Time PCR System (Applied Biosystems, Foster City, CA, USA).

## Results and discussion

### Pathway engineering to reduce byproduct formation

For removing pathogenicity of the strain, we deleted *wabG* gene from *K. pneumoniae* KCTC 2242, which is responsible for making a lipopolysaccharide [26]. The resulting strain, KMK-01 is a parental strain in this study. *K. pneumoniae* KMK-01 produces 1,3-PDO as a major product from glycerol. At the same time, it also produces a significant number of byproducts, including 2,3-BDO, lactate, acetate, and succinate (Table 3). Deletions of *ldhA* and *pflB* have been reported significantly reducing the amounts of these byproducts when glucose is fed as a carbon source [12]. When KMK-02, the KMK-01 Δ*ldhA* mutant, was cultured in glycerol, lactate production was reduced from 0.06 to 0 mol mol^−1^. KMK-05, the KMK-02 Δ*pflB* mutant, produced no acetate, similar to previous results observed for glucose [12]. 1,3-PDO production increased from 17.94 to 20.14 g L^−1^ and 20.11 g L^−1^ in KMK-02 and KMK-05, respectively, compared to the parental strain, while the molar yield from glycerol was almost the same. Meanwhile, 2,3-BDO production increased in these mutants. Therefore, *budA* encoding 2,3-butanediol dehydrogenase, was deleted from the KMK-05 strain and the resulting strain was designated KMK-12. KMK-12 produced less 2,3-BDO than the parental strain, 0.04 compared to 0.23 mol mol^−1^, respectively. Despite the reduction in byproduct formation by KMK-12, the molar yield of 1,3-PDO from glycerol, 0.47 mol mol^−1^ glycerol, did not notably change (Table 3). Meanwhile, deletion of *budA* caused significant decreases in glycerol consumption and biomass production, resulting in a reduction in 1,3-PDO titer, as previously reported [13].

**Table 3:** OD_600_, glycerol consumption and metabolite production yields in *K. pneumoniae* wild type and engineered strains after 24 h of flask culture^a^

*ldhA*		Δ	Δ	Δ
*pflB*			Δ	Δ
*budA*				Δ

Strain name	KMK-01	KMK-02	KMK-05	KMK-12
OD_600_	9.66	12.18	6.47	3.65
Consumed glycerol (g L^−1^)	48.31	54.45	52.55	16.13
Acetate production (g L^−1^)	1.42	1.51	0.00	1.34
Succinate production (g L^−1^)	0.87	0.84	0.82	0.36
Lactate production (g L^−1^)	2.89	0.00	0.59	0.00
2,3-BDO production (g L^−1^)	6.00	6.65	11.57	0.60
1,3-PDO production (g L^−1^)	17.94	20.14	20.11	6.30
1,3-PDO productivity (g L^−1^ h^−1^)	0.75	0.84	0.84	0.26
1,3-PDO yield (mol mol^−1^)^b^	0.45	0.45	0.46	0.47

^a^ Experiments were repeated three times independently

^b^ Yield was calculated as produced metabolite (mol)/consumed glycerol (mol)

### Elimination of glycerol assimilation pathways to improve 1,3-PDO yield

**Fig. 2:**
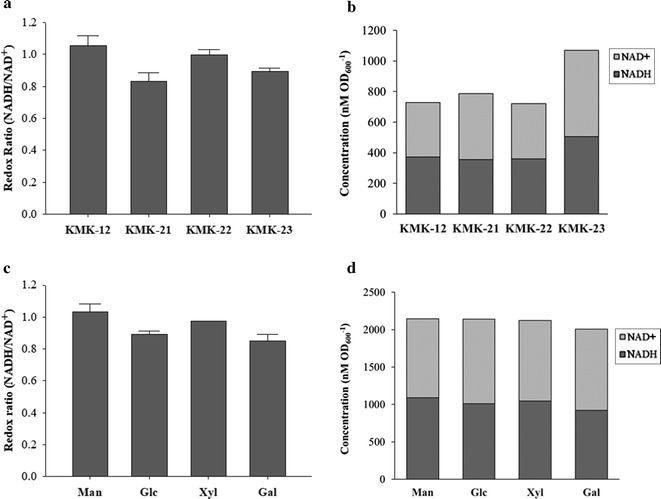
**a** NADH/NAD^+^ ratios and **b** NADH and NAD^+^ concentrations of KMK-12, KMK-21, KMK-22 and KMK-23 strains grown in 55 g L^−1^ glycerol medium. **c** NADH/NAD^+^ ratios and **d** NADH and NAD^+^ concentrations of KMK-23 grown in the medium containing 40 g L^−1^ glycerol and 20 g L^−1^ various co-substrates; mannitol (Man), glucose (Glc), xylose (Xyl) and galactose (Gal)

To improve the molar yield of 1,3-PDO from glycerol, glycerol assimilation pathways were eliminated on *K. pneumoniae*. Two glycerol assimilation pathways, which start from glycerol and lead into glycolysis, exist in *K. pneumoniae* (Fig. 1a). Glycerol kinase (GlpK, NCBI-Protein ID: AEK00501) is responsible for the conversion of glycerol to glycerol 3-phosphate, while glycerol dehydrogenase (DhaD, NCBI-Protein ID: AEJ99991) converts glycerol to dihydroxyacetone with NADH production [27–29]. GlpK is active under aerobic conditions, while DhaD is active under anaerobic conditions [29]. Strains KMK-21 and 22 were constructed by deleting *dhaD* and *glpK*, respectively, from KMK-12. Strain KMK-23 was generated by deleting both *dhaD* and *glpK* from KMK-12. When these strains were cultured for 24 h in flasks containing glycerol, there were decreases in 1,3-PDO productions and molar yields in KMK-12 and KMK-23, while a marginal increase in 1,3-PDO production was in KMK-22 (Additional file 3: Table S2). It is because DhaD–DhaKLM pathway provides 1 mol of NADH for 1,3-PDO production, while GlpK–GlpD pathway does not (Fig. 1a). Therefore, *dhaD* deletion resulted in decreases in 1,3-PDO production, while *glpK* deletion did not. To prove this, NADH/NAD^+^ ratios were measured in KMK-12, KMK-21, KMK-22, and KMK-23 strains. The results also supported that the strains with *dhaD* deletion, KMK-21 and KMK-23 produced less NADH, resulting in low NADH/NAD^+^ ratios (Fig. 2a and b).

To enhance 1,3-PDO yield, glycerol flux towards assimilation should be restricted further. Therefore, glucose was supplied as co-substrate to replace the glycolytic flux generated from glycerol assimilation. When glycerol and glucose were supplied together, the molar yield of 1,3-PDO from glycerol in KMK-21 and KMK-23 significantly increased (Table 4). Since glucose was not converted to 1,3-PDO (Additional file 4: Table S3), the molar yield of 1,3-PDO was calculated based on glycerol consumed. Deletion of *dhaD* (strain KMK-21) improved molar yield from 0.56 to 0.77 mol mol^−1^ compared to the parental strain (KMK-12). When *dhaD* was deleted, glycerol consumption was restricted because DhaD is active under microaerobic conditions and, therefore, this mutant assimilated very little glycerol. Deletion of *glpK* (KMK-22) improved 1,3-PDO production from 4.87 to 6.09 g L^−1^ compared to KMK-12, with a marginal improvement in yield. This may be because deletion of *glpK* enhances DhaD activity by more than twofold according to Ashok et al. [30]. However, it also enhances the flux into glycerol assimilation pathways, draining glycerol for energy and cell mass production. Therefore, no gain in 1,3-PDO molar yield from glycerol was observed for KMK-22. When both the *glpK* and *dhaD* genes were deleted, the molar yield of 1,3-PDO from glycerol improved from 0.56 to 0.84 mol mol^−1^ glycerol, with an improvement in titer from 4.87 to 5.27 g L^−1^. This implies that elimination of glycerol assimilation enhanced carbon flux from glycerol towards 1,3-PDO production, while energy and cell mass production were supported by flux from carbon to glucose. Compared to the previously reported molar yield of 1,3-PDO from glycerol, 0.60 mol mol^−1^ glycerol, [15, 16], the molar yield obtained by KMK-23 was much higher, 0.84 mol mol^−1^ glycerol. Despite that, we observed increased acetate production from engineered strains. Acetate production could be reduced by keeping glycerol or glucose concentration low during fed-batch fermentation [8, 31].

**Table 4:** Comparison of OD_600_, glycerol consumption, 1,3-PDO production and yield in KMK-12 and its mutants after 24 h of flask culture^a^

*dhaD*		Δ		Δ
*glpK*			Δ	Δ

Strain name	KMK-12	KMK-21	KMK-22	KMK-23
OD_600_	3.53	3.26	3.32	3.85
Consumed glycerol (g L^−1^)	10.49	7.49	13.56	7.59
Consumed glucose (g L^−1^)	7.80	9.24	7.15	11.97
Acetate production (g L^−1^)	0	2.25	1.47	1.78
Succinate production (g L^−1^)	0.23	0.14	0.20	0.25
1,3-PDO production (g L^−1^)	4.87	4.79	6.09	5.27
1,3-PDO productivity (g L^−1^ h^−1^)	0.20	0.20	0.25	0.22
1,3-PDO yield (mol mol^−1^)^b^	0.56	0.77	0.54	0.84

^a^ Experiments were repeated three times independently

^b^ Yield is calculated as dividing produced metabolite by consumed glycerol

### Selecting a co-substrate for KMK-23

**Fig. 3:**
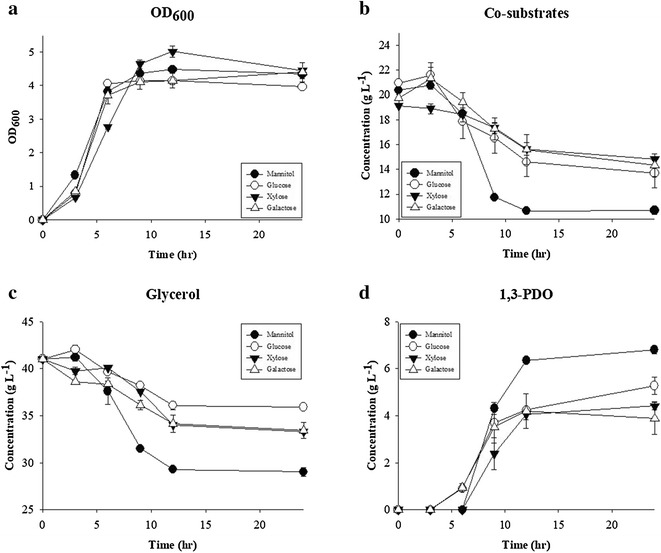
**a** Microbial growth (OD_600_), **b** consumption of co-substrates, **c** consumption of glycerol, and **d** 1,3-PDO production of KMK-23 in flask culture in the medium containing 40 g L^−1^ glycerol and 20 g L^−1^ four different co-substrates; mannitol (filled circle), glucose (empty circle), xylose (filled reversed triangle), and galactose (empty triangle)

After eliminating glycerol assimilation pathways, co-substrates, i.e., glucose alternatives, were screened for efficient 1,3-PDO production. Mannitol, glucose, galactose, and xylose were tested as co-substrates with glycerol (Fig. 3). Out of the four co-substrates tested, mannitol resulted in the highest 1,3-PDO production titer, 7.84 g L^−1^, which is 48.7% more than that of glucose (Fig. 3). Comparing to the other three co-substrates, mannitol utilization pathway produces additionally 1 mol of NADH when mannitol-1-phosphate converts to fructose-6-phosphate [32, 33]. Indeed, NADH/NAD^+^ ratio was highest when mannitol was used as co-substrate (Fig. 2c, d). It is suspected that this additional NADH was beneficial to 1,3-PDO production, since 1,3-PDO production requires NADH for DhaT (NCBI-Protein ID: AEJ99987) enzymatic reaction (Fig. 2c, d).

**Fig. 4:**
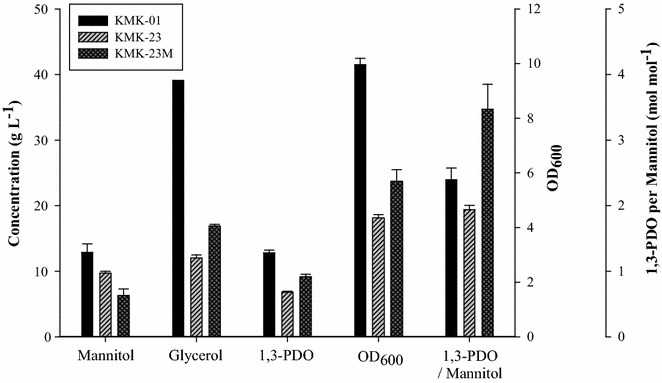
Effects of genome editing on 5′-UTR of *mtlA* gene for its reduced expression on carbon source consumption and metabolite productions. When three strains, KMK-01 (wild type, black bar), KMK-23 (dashed bar), and KIK-23M (genome-edited strain, gray bar), were cultured in the medium containing 40 g L^−1^ glycerol and 20 g L^−1^ mannitol, mannitol and glycerol consumption, 1,3-PDO production, microbial growth (OD_600_), and 1,3-PDO production per unit mannitol consumption for 24 h were compared

However, when mannitol was used as a co-substrate, its consumption over 24 h was much higher than consumption of other co-substrates (Fig. 3). To reduce fermentation cost, mannitol consumption should be restricted. Here, we attempted to solve it by decreasing the expression level of mannitol-specific transporter, MtlA (NCBI-Protein ID: AEK00431) by genome editing [32–34]. The 5′-UTR sequence of the *mtlA* was determined by RBS calculator and then was edited by CRISPR/Cas9 method as described in “Methods”. The resulting strain was confirmed by sequencing and designated as KMK-23M. The expression level of *mtlA* in KMK-23M was decreased 25% compared to KMK-23 (Additional file 5: Figure S2A). Mannitol consumption of KMK-23M until 24 h was reduced from 9.68 to 6.28 g L^−1^ compared to that of KMK-23, while cell mass and 1,3-PDO production both increased substantially by 31 and 34%, respectively (Fig. 4). No reduction was observed in molar yield of 1,3-PDO from glycerol. This suggests that for KMK-23, the glycolytic flux supplied by mannitol exceeded the optimal point. Therefore, reducing glycolytic flux in KMK-23M benefited both cell growth and the conversion of glycerol to 1,3-PDO.

### Engineering of transcriptional regulation of the *dha* operon

Figure 1b presents the arrangement of the *dha* genes in the *K. pneumoniae* genome. The genes encoding the enzymes are directly involved in 1,3-PDO production, *dhaB* (Gene ID: 12547480 [Genbank]) and *dhaT* (Gene ID: 12547484 [Genbank]), which belong to the *dha* operon [14]. According to a study by Bächler et al. [35], the promoter of the *dhaKLM* operon in *Escherichia coli*, PdhaK, is negatively regulated by DhaK and DhaM, and positively regulated by DhaL. Dephosphorylated DhaL can bind the sensing domain of DhaR (NCBI-Protein ID: AEJ99990) and activate DhaR. DhaR positively regulates *dhaT* expression, but negatively regulates *dhaB* [36]. Negative regulation of *dhaB* could be beneficial to 1,3-PDO production because the product of DhaB, 3-hydroxypropionaldehyde, is a toxic intermediate [37]. DhaR is repressed by DhaK, while activated by dephosphorylated DhaL that is inactivated by DhaM-mediated phosphorylation [35, 38]. In other words, *dhaK* and *dhaM* negatively regulate and *dhaL* positively regulates *dhaT* expression via DhaR.

**Fig. 5:**
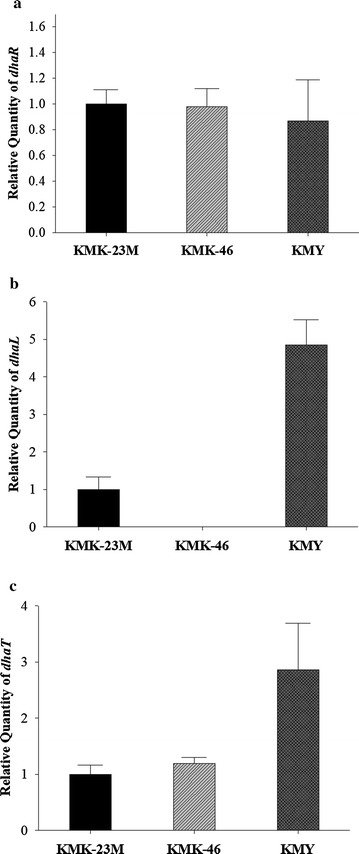
Relative expression levels of **a** *dhaR*, **b** *dhaL* and **c** *dhaT* in KMK-23M, KMK-46 and KMY strains detected by real-time RT-PCR. The expression levels of KMK-46 and KMY were normalized with cell mass (OD_600_) and with the ones of control strain, KMK-23M. The expression levels were measured three times with the strains grown in 40 g L^−1^ glycerol and 20 g L^−1^ mannitol up to mid-log phase and the error bars represented the standard deviations

**Fig. 6:**
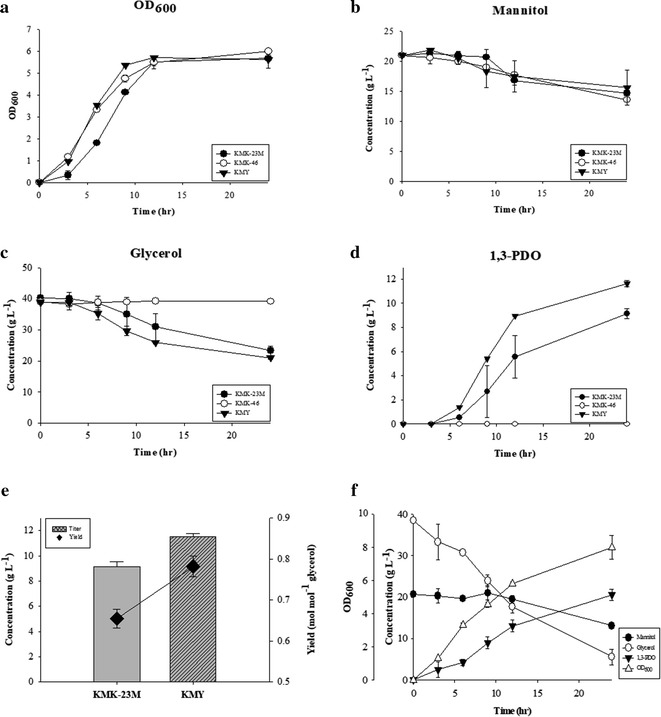
**a** Microbial growth (OD_600_), **b** mannitol, **c** glycerol, and **d** 1,3-PDO concentrations of KMK-23M (filled circle), KMK-46 (empty circle), and KMY (filled reversed triangle) strains in flask culture. **e** Comparison of 1,3-PDO production (g L^−1^) and its molar yield (mol mol^−1^ glycerol) between KMK-23M and KMY strains during 24 h. Bars represent titer and diamond-shaped dot represent yields. **f** Batch fermentation results of KMY strain with the concentrations of mannitol (filled circle) and glycerol (empty circle), 1,3-PDO (filled reversed triangle), and OD_600_ (empty triangle)

We deleted *dhaKLM* on KMK-23M strain genome and named the resulting strain KMK-46. Then, the *dhaL* gene-encoding activator was overexpressed and the resulting strain was designated KMY, expecting DhaR activated by DhaL transcriptionally upregulates *dhaT* expression (Fig. 1b). To demonstrate the expression level changes of *dhaR*, *dhaL* and *dhaT* quantitative RT-PCR was conducted in KMK-23M, KMK-46, and KMY strains. The expression levels of *dhaR* were not changed significantly in those engineered strain (Fig. 5a). On the other hand, *dhaL* expression levels confirmed that *dhaKLM* deletion and *dhaL* overexpression were properly conducted in KMK-46 and KMY strains (Fig. 5b). As expected, *dhaT* expression in KMY was increased threefold than KMK-23M and KMK-46 (Fig. 5c). Interestingly, KMK-46, *dhaKLM* deleted strain, did not produce 1,3-PDO at all, while *dhaL* overexpression in that strain increased 1,3-PDO production by 26.4% compared to KMK-23M (Fig. 6a–d). The molar yield of KMY was also 20% higher than for KMK-23M (Fig. 6e). This is contrast to the result of overexpression of *dhaR* (Additional file 5: Figure S2B) in the KMK-46 strain, resulted in no 1,3-PDO production (data not shown). In addition, when *dhaT* was overexpressed in KMK-23M and KMK-46 strains, 1,3-PDO production was much less than that of KMY (data not shown). These results suggest that the regulation of *dha* operon is not simple and has not been fully understood yet. In this study, the developed KMY strain with deletion of *dhaKLM* and overexpression of *dhaL* partially proved their regulatory functions on *dhaT* expression.

Batch fermentation was performed with the resulting strain, KMY. A total of 20.59 g L^−1^ 1,3-PDO was produced in 24 h with a molar yield of 0.76 mol mol^−1^ glycerol (Fig. 6f). The 1,3-PDO yield from the carbon sources in total, including mannitol, was 0.54 g g^−1^. The molar yield in this study was superior to that reported in other published studies; these studies reported molar yields of around 0.6 mol mol^−1^ glycerol [15, 16]. The increase in the 1,3-PDO titer from batch fermentation compared to culturing in flasks may be due to the pH control during the process. During the entire fermentation, dissolved oxygen level was maintained at near zero percent, because the microaerobic condition with low aeration has been known to be beneficial for 1,3-PDO production [5]. The developed strain produced significantly lower amounts of byproducts with a much higher molar yield of 1,3-PDO from glycerol. This could be extremely helpful in reducing the cost of product separation [11]. The high-molar yield of 1,3-PDO was enabled by eliminating genes in the glycerol assimilation pathway and feeding bacteria a co-substrate for cell mass and maintenance energy. Regulators influencing expression of *dhaT* were also helpful in increasing 1,3-PDO production and yield compared to the parental strain. With continuous metabolic engineering, a cost-effective biochemical process to produce 1,3-PDO using *K. pneumoniae* would be possible near future.

## Conclusions

In this study, we minimized the production of byproducts during 1,3-PDO synthesis by deleting the pathway genes from the *K. pneumoniae* genome. Next, the glycerol assimilation pathway was eliminated and mannitol was fed as a co-substrate to improve glycerol flux towards 1,3-PDO production. The 5′-UTR of *mtlA* was edited to reduce mannitol consumption. Finally, 1,3-PDO production was enhanced by modifying *dha* regulation through deletion of *dhaKLM* and overexpression of *dhaL*. In batch fermentation, 1,3-PDO was produced with the yield of 0.76 mol mol^−1^ glycerol. The molar yield in this study was superior to previously published studies.

## Additional file

**Additional file 1. Table S1:** Oligonucleotides used in this study.

**Additional file 2. Figure S1:** Confirmation experiments for deletion mutants. **A** PCR fragments from genomic DNA of *K. pneumoniae* KCTC 2242, KMK-01, KMK-02 and KMK-05 for *wabG*, *ldhA*, *pflB*, *budA* sites. **B** PCR fragments from KMK-12, KMK-21, KMK-22 and KMK-23 for *dhaD* and *glpK* sties. **C** PCR fragments from KMK-23M and KMK-46 for *dhaKLM* site. The genes from control strains are written in red, while ones from deleted strains are written in light blue.

**Additional file 3. Table S2:** Fermentation data of KMK-12 and its mutants after 24 hrs of flask cultivation with 40 g L^−1^ glycerol as a sole carbon source.

**Additional file 4. Table S3:** Fermenation data of KMK-12 and its mutants after 24 hrs of flask cultivation with 40 g L^−1^ glucose as a sole carbon source.

**Additional file 5. Figure S2:** Relatively gene expression levels of **A** *mtlA* in KMK-23 and KMK-23M when glucose or mannitol was used as a co-substrate and **B** *dhaR* when the gene was overexpressed in KMK46 strain. The gene expression levels were detected by quantitative RT-PCR.

## Abbreviations

1,3-PDO: 1,3-propanediol
2,3-BDO: 2,3-butanediol
BudA: alpha-acetonelactate decarboxylase
DhaB: glycerol dehydratase
DhaD: glycerol dehydrogenase
DhaK: dihydroxyacetone kinase subunit K
DhaL: dihydroxyacetone kinase subunit L
DhaM: dihydroxyacetone kinase subunit M
DhaR: DNA-binding transcriptional regulator of *dha* operon
DhaT: 1,3-PDO dehydrogenase
GlpK: glycerol kinase
LdhA: lactate dehydrogenase
MtlA: mannitol-specific transporter
PdhaK: *dhaK* promoter
PflB: formate acetyltransferase
