# Supplementary material for: High-yield production of 1,3-propanediol from glycerol by metabolically engineered Klebsiella pneumoniae
Source: Biotechnol Biofuels. 2018 Apr 9;11:104. doi: 10.1186/s13068-018-1100-5 (PMC5890353; doi:10.1186/s13068-018-1100-5)
Supplement: Supplementary file 1 — Additional file 1. Table S1: Oligonucleotides used in this study. [file 13068_2018_1100_MOESM1_ESM.docx]

**Table S1.** Oligomers used in this study.

| Name | Sequence (5'-3') | Source |
| --- | --- | --- |
| Oligomer |  |  |
| wabG con A | GGACGCTATCAACACCAACA | [12] |
| wabG con B | ATGGGTACTCGCAGACATCG | [12] |
| ldhA con A | GAATTAGGATTAGCACCCTCTCA | [12] |
| ldhA con B | CCAAGCCAGTGTAACGGTATC | [12] |
| pflB con A | GGGTTGACATACTGGGTCATTT | [12] |
| pflB con B | GCGTCGAGTCTGTTTTGACA | [12] |
| budA F | **GTCAACATTTATTTAACCTTTCTTATATTTGTTGAACGAGGAAGTGGTAT**GTGTAGGCTGGAGCTGCTTC | This study |
| budA R | **GCGCCGTGCGCCCACTGGCGTACCGGATACTGTTTGTCCATGTGACCCCC**GTCCATATGAATATCCTCCT | This study |
| budA con A | ACGGAGGCTGTGAAATACCC | This study |
| budA con B | TTCGTGGCGTACCGGAATA | This study |
| dhaD F | **ACGCCAGGGCTCATCATGTCTACATGCGCACTTATTTGAGGGTGAAAGGA**GTGTAGGCTGGAGCTGCTTC | This study |
| dhaD R | **GGGGAGAAAGAAAAACCGCTGACCTGGGCCAGCGGTTTAGCCACCGCGAA**GTCCATATGAATATCCTCCT | This study |
| dhaD con A | AAAAATTAACCTGTGTTTCATATCAG | This study |
| dhaD con B | GTTTCGCGCTGCATAAACTT | This study |
| glpK F | CCACCGCTCAACATAAAGCTTCGCTGTAATCTGACTACGGGACACCGACTGTGTAGGCTGGAGCTGCTTC | This study |
| glpK R | GTAGCCCGGCTAAGGCGTTTACGCCGCAAGCCGGGAATAACTTCACAATCGTCCATATGAATATCCTCCT | This study |
| glpK con A | GGCGGCAAAGATATTCCTTA | This study |
| glpK con B | GTTTCACCGCTTTCTTCCAG | This study |
| glpK con C | CGAAGAACTCCAGCATGAGA | This study |
| mtlA CRISPR F | AAACAAGCCTGCGCGCTACGCAAAACAGAAGAAGG | This study |
| mtlA CRISPR R | TTTTGGAAGAAGACAAAACGCATCGCGCGTCCGAA | This study |
| mtlA rescue F | CCGGCTACCTCTGCCGCCACATTAACAACAAACCTCGGGCTTTAAGCCTGTAGACAGAGTCTAACAGACCATCGAG | This study |
| mtlA rescue R | CTGAGGAAACGACCAAAGCTTTGCACTTTGATCTTGATATCGGATGACATACGTTCCTCGATGGTCTGTTAGACTC | This study |
| dhaKLM F | **TCCGCCAGCAGAGAGGCGGCATCGAACCTTCTCCAACGGAGCACTGAATA**GTGTAGGCTGGAGCTGCTTC | This study |
| dhaKLM R | **ACTCACCCTCCGGAGGCAGGCCCCCGGAGCGGTAACGGTCAACGTGAATT**GTCCATATGAATATCCTCCT | This study |
| dhaKLM con A | AAAACGTTCCATAAAGAAACAAAA | This study |
| dhaKLM con B | GGTTCCTGAAGAGTAAGCGAAA | This study |
| dhaL F | **CGAGGTCGACGGTATCGATA**ATGTCACTGAACAGAACGCAAATC | This study |
| dhaL R | **TTTATTTGATGCCTCTAGCA**TTACTCTTTGGCGGCGGC | This study |
| RT gapA F | CGTGAAACTGGTTTCCTGGT | This study |
| RT gapA R | AGATGTGGGCAATCAGATCC | This study |
| RT dhaR F | CCGGGAAGGCGGTCTCT | This study |
| RT dhaR R | AGGTTTCGCGCTGCATAAA | This study |
| RT dhaL F | CGACCACGGTCTCAATATGC | This study |
| RT dhaL R | CGAATGAAGAAGGTGCCGAA | This study |
| RT dhaT F | GGAATCGAGACCCTGACCAA | This study |
| RT dhaT R | CAGCAGCGGATCGTTGATAG | This study |
| RT mtlA F | GCGCGGATAACATCTTCCTC | This study |
| RT mtlA R | TCAACGTAACCGCCTTTCAC | This study |
