# Supplementary material for: High-yield production of 1,3-propanediol from glycerol by metabolically engineered Klebsiella pneumoniae
Source: Biotechnol Biofuels. 2018 Apr 9;11:104. doi: 10.1186/s13068-018-1100-5 (PMC5890353; doi:10.1186/s13068-018-1100-5)
Supplement: Supplementary file 2 — Additional file 2. Figure S1: Confirmation experiments for deletion mutants. A PCR fragments from genomic DNA of K. pneumoniae KCTC 2242, KMK-01, KMK-02 and KMK-05 for wabG, ldhA, pflB, budA sites. B PCR fragments from KMK-12, KMK-21, KMK-22 and KMK-23 for dhaD and glpK sties. C PCR fragments from KMK-23M and KMK-46 for dhaKLM site. The genes from control strains are written in red, while ones from deleted strains are written in light blue. [file 13068_2018_1100_MOESM2_ESM.docx]

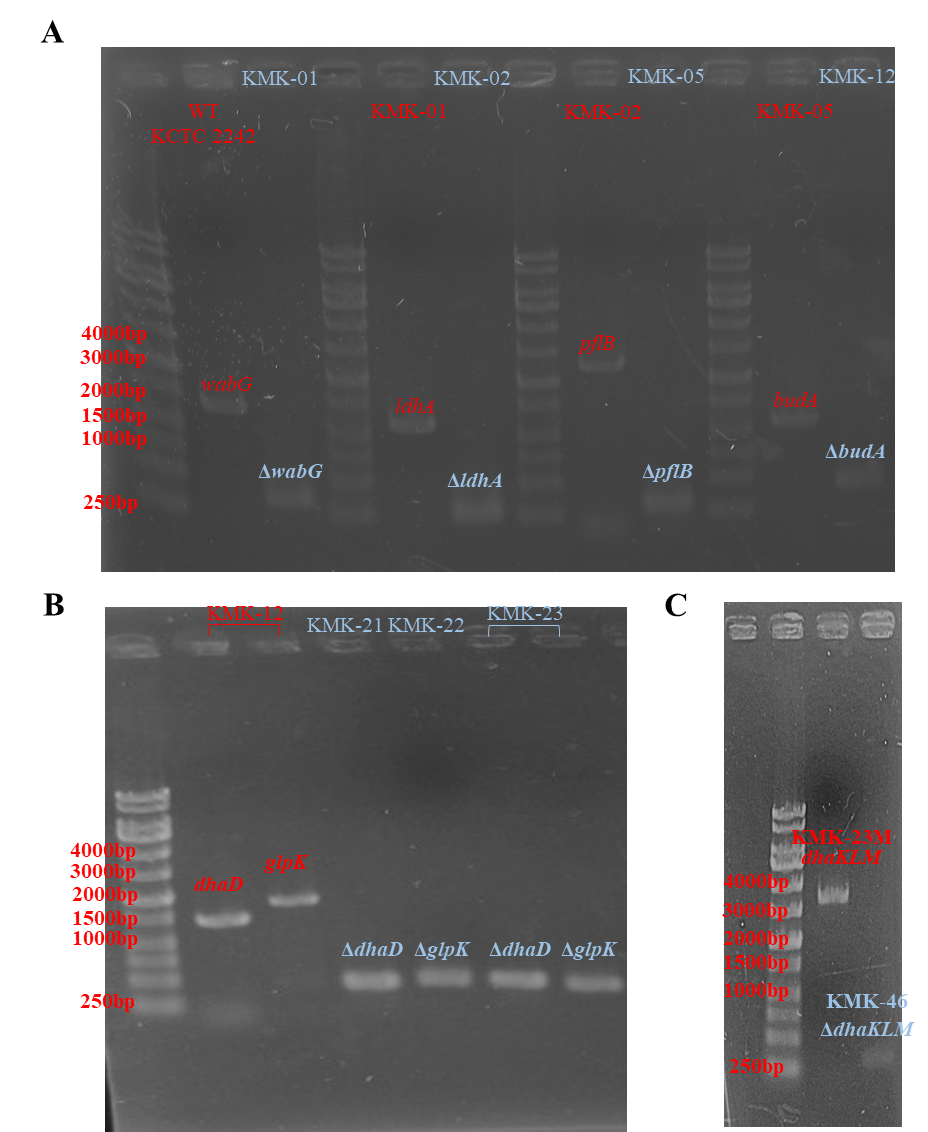


**Figure S1.** Confirmation PCR experiments for deletion mutants. **A.** PCR fragments from genomic DNA of *K. pneumoniae* KCTC 2242, KMK-01, KMK-02 and KMK-05 for *wabG*, *ldhA*, *pflB*, *budA* sites. **B.** PCR fragments from KMK-12, KMK-21, KMK-22 and KMK-23 for *dhaD* and *glpK* sties. **C.** PCR fragments from KMK-23M and KMK-46 for *dhaKLM* site. The genes from control strains are written in red, while ones from deleted strains are written in light blue.
