# Supplementary material for: High-yield production of 1,3-propanediol from glycerol by metabolically engineered Klebsiella pneumoniae
Source: Biotechnol Biofuels. 2018 Apr 9;11:104. doi: 10.1186/s13068-018-1100-5 (PMC5890353; doi:10.1186/s13068-018-1100-5)
Supplement: Supplementary file 3 — Additional file 3. Table S2: Fermentation data of KMK-12 and its mutants after 24 hrs of flask cultivation with 40 g L−1 glycerol as a sole carbon source. [file 13068_2018_1100_MOESM3_ESM.docx]

**Table S2.** Comparison of OD_600_, glycerol consumption, 1,3-PDO production and yield in KMK-12 and its mutants after 24 hrs of flask cultivation. 40 g L^-1^ glycerol was used as a sole carbon source with rich medium components

| *dhaD* |  | Δ |  | Δ |
| --- | --- | --- | --- | --- |
| *glpK* |  |  | Δ | Δ |
| Strain name | KMK-12 | KMK-21 | KMK-22 | KMK-23 |
| OD_600_ | 3.26 | 4.72 | 3.42 | 4.32 |
| Glycerol uptake (g L^-1^) | 16.13 | 11.88 | 21.35 | 14.06 |
| Acetate production (g L^-1^) | 1.34 | 3.51 | 2.15 | 2.84 |
| Succinate production (g L^-1^) | 0.36 | 0.03 | 0.06 | 0.01 |
| 1,3-PDO production (g L^-1^) | 6.30 | 3.68 | 7.17 | 4.37 |
| 1,3-PDO yield (mol mol^-1^) | 0.47 | 0.38 | 0.41 | 0.38 |
