# Supplementary material for: High-yield production of 1,3-propanediol from glycerol by metabolically engineered Klebsiella pneumoniae
Source: Biotechnol Biofuels. 2018 Apr 9;11:104. doi: 10.1186/s13068-018-1100-5 (PMC5890353; doi:10.1186/s13068-018-1100-5)
Supplement: Supplementary file 4 — Additional file 4. Table S3: Fermenation data of KMK-12 and its mutants after 24 hrs of flask cultivation with 40 g L−1 glucose as a sole carbon source. [file 13068_2018_1100_MOESM4_ESM.docx]

**Table S3.** Comparison of OD_600_, glucose consumption, 1,3-PDO production and yield in KMK-12 and its mutants after 24 hrs of flask cultivation. 40 g L^-1^ glucose was used as a sole carbon source with rich medium components

| *dhaD* |  | Δ |  | Δ |
| --- | --- | --- | --- | --- |
| *glpK* |  |  | Δ | Δ |
| Strain name | KMK-12 | KMK-21 | KMK-22 | KMK-23 |
| OD_600_ | 4.53 | 3.74 | 4.55 | 3.92 |
| Glucose uptake (g L^-1^) | 19.31 | 18.42 | 19.37 | 17.83 |
| 1,3-PDO production (g L^-1^) | 0 | 0 | 0 | 0 |
| Acetate production (g L^-1^) | 0.09 | 0.89 | 0.38 | 0.91 |
| Succinate production (g L^-1^) | 0.15 | 0.13 | 0.25 | 0.09 |
