# Supplementary material for: High-yield production of 1,3-propanediol from glycerol by metabolically engineered Klebsiella pneumoniae
Source: Biotechnol Biofuels. 2018 Apr 9;11:104. doi: 10.1186/s13068-018-1100-5 (PMC5890353; doi:10.1186/s13068-018-1100-5)
Supplement: Supplementary file 5 — Additional file 5. Figure S2: Relatively gene expression levels of A mtlA in KMK-23 and KMK-23M when glucose or mannitol was used as a co-substrate and B dhaR when the gene was overexpressed in KMK46 strain. The gene expression levels were detected by quantitative RT-PCR. [file 13068_2018_1100_MOESM5_ESM.docx]

**A**


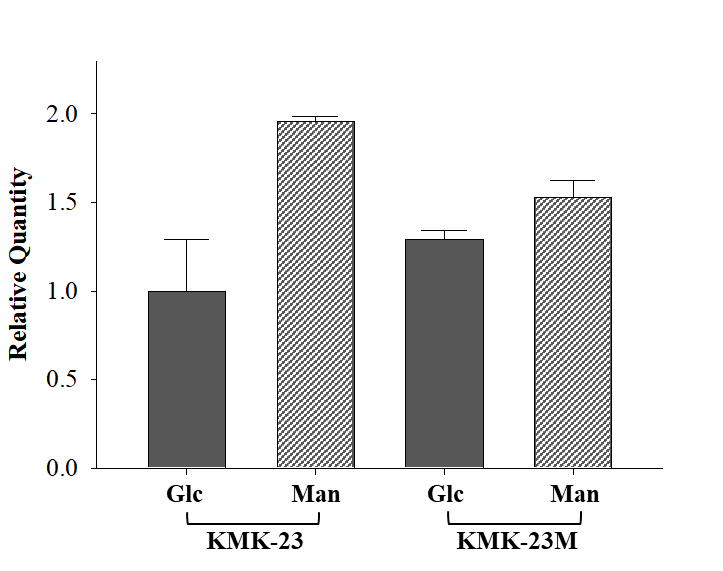


**B**


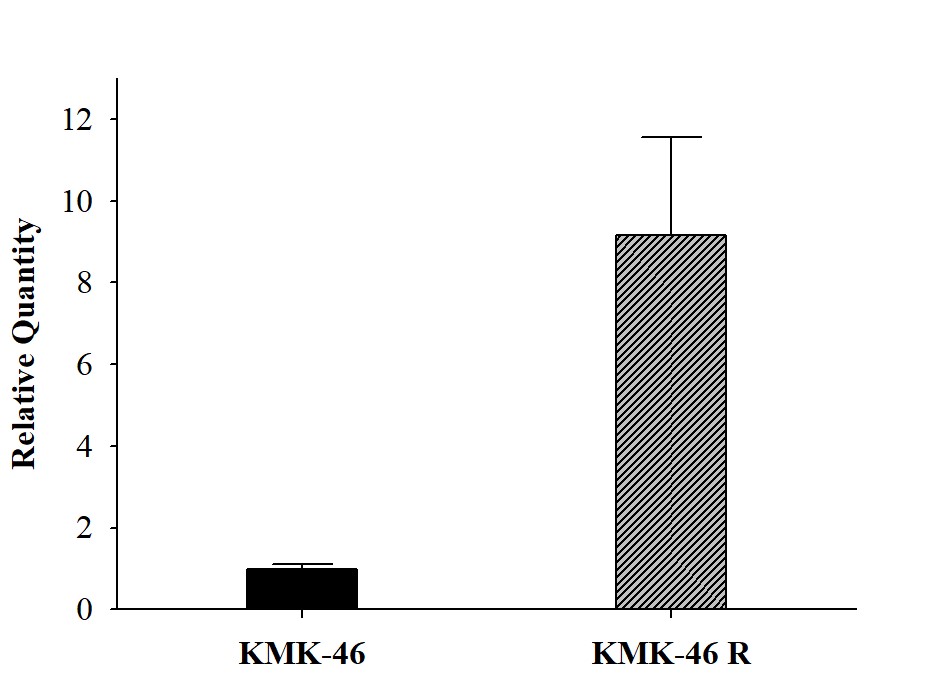


**Figure S2.** Relatively gene expression levels of **A.** *mtlA* in KMK-23 and KMK-23M when glucose or mannitol was used as a co-substrate and **B.** *dhaR* when the gene was overexpressed in KMK46 strain. The gene expression levels were detected by quantitative RT-PCR.
